# Primary splenic multicystic peritoneal mesothelioma in a young healthy male

**DOI:** 10.1093/jscr/rjad551

**Published:** 2023-10-14

**Authors:** Matthew D Price, Shuait Nair, James Harris

**Affiliations:** Department of Surgery, Johns Hopkins School of Medicine, 1800 Orleans Street, Baltimore, MD 21287, United States; The Johns Hopkins University School of Medicine, Baltimore, MD 21287, United States; Department of Surgery, Johns Hopkins School of Medicine, 1800 Orleans Street, Baltimore, MD 21287, United States; Department of Surgery, The Johns Hopkins Howard County General Hospital, Howard County, MD 21044, United States

**Keywords:** benign multicystic peritoneal mesothelioma, spleen, splenic, cytokeratin

## Abstract

Multicystic peritoneal mesothelioma (BMPM) is a rare, usually benign tumor that arises from peritoneal mesothelial cells that most commonly occurs in women of reproductive age. Pathogenesis of these tumors is thought to come from chronic inflammation from prior surgery, endometriosis, trauma, or recurrent peritonitis. Here we report a case of primary splenic BMPM in a 20-year-old male with no past medical or surgical history and without any typical risk factors for this condition. He underwent an open splenectomy without complication. Pathology revealed an 18 × 4 × 11 cm^3^ spleen with a cyst occupying 75% of the splenic surface. Sections revealed a multilocular cyst with trabeculated walls and immunohistochemical staining positive for cytokeratin (AE1/AE3) consistent with BMPM. One year post operatively he remains asymptomatic; however, his interval computed tomography (CT) scan revealed several sub centimeter nodules that either represents small splenules or neoplastic implants. These will be followed with close interval imaging.

## Introduction

Multicystic peritoneal mesothelioma (BMPM) is a rare, usually benign tumor that arises from peritoneal mesothelial cells, with just over 200 cases reported in the literature [1]. BMPM usually occurs in females of reproductive age, though there have been several reported occurrences in men [1–5]. The etiology and pathogenesis of these tumors is debated, but the current working theory is that chronic irritation, either from previous surgery, endometriosis, recurrent peritonitis, or trauma can lead to mesothelial cell entrapment and reactive proliferation [1, 6–8]. Though these tumors are considered benign there is a high rate of local recurrence with a few reports of malignant transformation [8, 9]. BMPM is not to be confused with the similarly named malignant peritoneal mesothelioma, which has a poor prognosis and is associated with asbestos exposure [8]. To date there has been only one reported case of BMPM of primary splenic origin, which was in a 58-year-old female [10]. Here we report a second case of primary splenic BMPM, this time in a 20-year-old male with no prior medical or surgical history and without a history of trauma.

## Clinical history

A 20-year-old male with no major past medical or surgical history presented for surgical evaluation after an abdominal computed tomography (CT) scan showed a 12.0 × 10.3 × 9.1 cm^3^ complex multilobulated septated cystic lesion in the upper pole of the spleen. He had initially presented to his primary care physician with 4 months of upper abdominal pain with no other symptoms such as nausea, vomiting, fevers, or shortness of breath. He denied any history of trauma, recent illness, or sick contacts. He had never traveled outside of the country and had been working in heating, ventilation, and air conditioning for 1 year.

**Figure 1:**
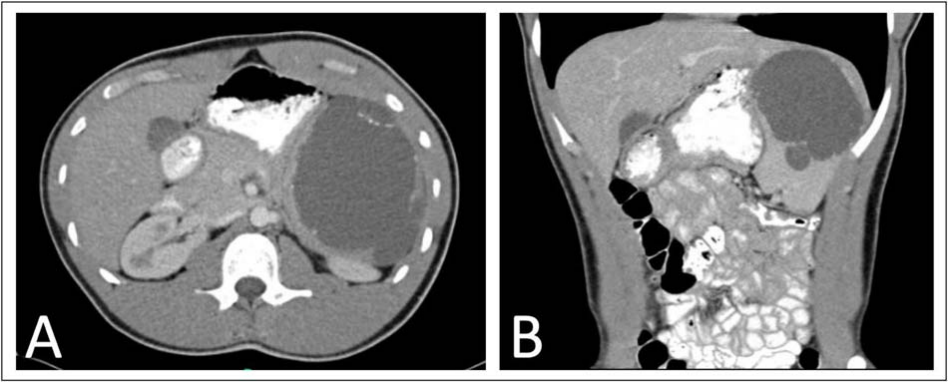
Preoperative (A) axial and (B) coronal abdominal CT images showing complex cystic splenic mass.

As part of his workup, he underwent an abdominal ultrasound showing a 12 cm cystic or hemorrhagic lesion, likely splenic in origin, with an adjacent 5.6 cm complex septated lesion within the spleen. Subsequent abdominal CT showed a 12 cm in greatest diameter complex cystic lesion within the spleen with peripheral and septal calcification with no solid enhancing component (Fig. 1). His blood work, including complete blood count, complete metabolic panel, and coagulation markers, were all within normal limits.

Given the size and unclear etiology of the lesion, he was scheduled for an open splenectomy. He was referred to infectious disease preoperatively for workup and for consideration of infectious etiologies, where he was started on albendazole for potential hydatid disease. Before surgery, he met with his primary care physician where he received the Menveo, Hib, and Prevnar 13 vaccines.

**Figure 2:**
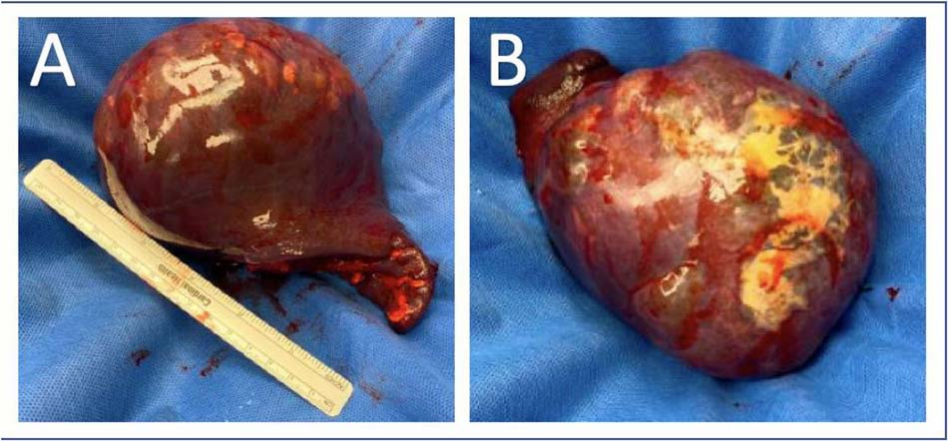
Splenectomy specimen with intact capsule with underlying cystic structure. (A) Anterior surface; (B) Posterior surface.

He was taken to the operating room and underwent an upper midline laparotomy. After inspection of the abdominal cavity and confirmation of no additional peritoneal lesions or significant adhesions to the spleen, the splenic vessels were controlled with 0-silk ties. The spleen was then mobilized and removed without violation of the splenic capsule and the abdomen closed (Fig. 2). He had an uncomplicated post-operative course and was discharged to home on post-operative Day 1.

Pathology revealed an 18.0 × 4.0 × 11.0 cm^3^ spleen weighing 855 g, with a 10.5 cm in greatest dimension cyst occupying 75% of the splenic surface. Sections revealed a multilocular cyst with copious cloudy yellow fluid and trabeculated walls. Immunohistochemical staining of the cyst wall was positive for cytokeratin (AE1/AE3) consistent with a benign multilocular mesothelial cyst without malignancy.

**Figure 3:**
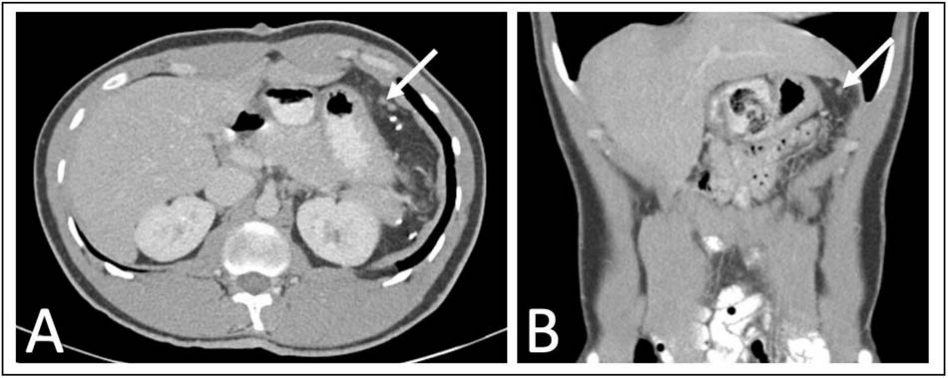
One year post operative abdominal CT scan (A) axial and (B) coronal abdominal CT images showing several sub-centimeter nodules in the post-splenectomy bed (arrows).

He was seen at 1, 6, and 12 months following surgery without complaints. A 1-year interval abdominal CT (Fig. 3) showed several 3–6 mm nodules in the left upper quadrant near the splenectomy site with the differential including several small lymph nodes, tiny splenules, or neoplastic implants that will be followed with another 1-year interval CT scan.

## Discussion and conclusions

Multicystic peritoneal mesothelioma is a rare condition with very few reported cases in men [2–5, 11, 12]. The predominant etiologic theory for BMPM is chronic inflammation can lead to reactive proliferation of mesothelial cells [6–8]. This is supported by many of the past reported cases, who have had prior surgery, or a history of endometriosis, PID, diverticulitis or other conditions resulting in chronic inflammation [1, 12]. Other etiologic theories include a neoplastic process, as a few cases have undergone malignant transformation, versus a hormonally driven process, given the female predominance and evidence of some tumors responses to anti-estrogen therapy [4, 13–15].

As BMPM is a benign process, symptoms usually occur once the tumor has grown sufficiently to cause mass effect on the surrounding organs or viscera, with non-specific abdominal complaints representing the most common presentation. Treatment includes complete surgical resection. Due to the estimated recurrence rate of 50% in women and 33% in men, some centers have elected to treat BMPM with complete cytoreduction followed by hyperthermic intraperitoneal chemotherapy (HIPEC) [4, 16, 17]. However, with a limited number of cases, this approach has not been adequately studied or compared with standard resection alone and regardless of treatment route, prognosis has been excellent [16, 17]. There are no published guidelines for post-operative follow-up, but given the high risk of recurrence, several centers recommend patients undergo an abdominal CT scan every 3-months for the first year followed by annual scans for the next 5 years [4, 18].

Presented here is a rare case of a splenic benign multicystic peritoneal mesothelioma in a 20-year-old male. With no prior surgical or medical history, the cause of BMPM in this patient does not fit the traditional etiological theory of chronic inflammation, highlighting a gap in our current understanding of the pathogenesis of BMPM. He was treated with an open splenectomy without HIPEC and at 1 year has recovered well with several sub-centimeter nodules in the splenectomy bed on CT that will be followed with an interval CT scan in 1 year.

## Data Availability

All data underlying the results are available as part of the article and no additional source data are required.
